# Recurrent human 16p11.2 microdeletions in type I Mayer–Rokitansky–Küster–Hauser (MRKH) syndrome patients in Chinese Han population

**DOI:** 10.1002/mgg3.2280

**Published:** 2023-10-03

**Authors:** Kaizhen Su, Han Liu, Xiaoqun Ye, Hangmei Jin, Zhenwei Xie, Chunbo Yang, Daizhan Zhou, Hefeng Huang, Yanting Wu

**Affiliations:** ^1^ The International Peace Maternity and Child Health Hospital School of Medicine Shanghai Jiao Tong University Shanghai China; ^2^ Shanghai Municipal Key Clinical Specialty Shanghai China; ^3^ Women's Hospital School of Medicine Zhejiang University Zhejiang China; ^4^ Bio‐X Institutes of Shanghai Jiao Tong University Shanghai China; ^5^ Obstetrics and Gynecology Hospital Institute of Reproduction and Development Fudan University Shanghai China; ^6^ Research Units of Embryo Original Diseases Chinese Academy of Medical Sciences (No. 2019RU056) Shanghai China

**Keywords:** 16p11.2 deletion, Chinese Han population, copy number variant, MRKH syndrome

## Abstract

**Backgrounds:**

Mayer‐Rokitansky‐Küster‐Hauser (MRKH) syndrome, a severe congenital malformation of the female genital tract, is a highly heterogeneous disease which has no clear etiology. Previous studies have suggested that copy number variations (CNVs) and single‐gene mutations might contribute to the development of MRKH syndrome. In particular, deletions in 16p11.2, which are suggested to be involved in several congenital diseases, have been reported in Chinese type II MRKH patients and European MRKH patients. However, few CNVs including 16p11.2 microdeletions were identified in Chinese type I MRKH cases although it accounted for the majority of MRKH patients in China. Thus, we conducted a retrospective study to identify whether CNVs at human chromosome 16p11.2 are risk factors of type I MRKH syndrome in the Chinese Han population.

**Methods:**

We recruited 143 patients diagnosed with type I MRKH between 2012 and 2014. Five hundred unrelated Chinese without congenital malformation were enrolled in control group, consisting of 197 from the 1000 Genomes Project and 303 from Fudan University. Quantitative PCR, array comparative genomic hybridization, and sanger sequencing were conducted to screen and verify candidate variant.

**Results:**

Our study identified recurrent 16p11.2 microdeletions of approximately 600 kb in two out of the 143 type I MRKH syndrome patients using high‐density array‐based comparative genomic hybridization (aCGH), while no 16p11.2 deletion was found in the control group. We did not find any mutations in *TBX6* gene in our samples.

**Conclusions:**

The results of the study identify 16p11.2 deletion in Chinese MRKH I patients for the first time, as well as support the contention that 16p11.2 microdeletions are associated with MRKH syndrome in both types across populations. It is suggested that 16p11.2 microdeletions should be included in molecular diagnosis and genetic counseling of female reproductive tract disorders.

## Graphical abstract

The study identified 16p11.2 micro deletions in Chinese type I MRKH syndrome patients for the first time, using qRT‐PCR and aCGH. However, Sanger sequencing did not identify any mutations in TBX6, a reported candidate causative gene located in 16p11.

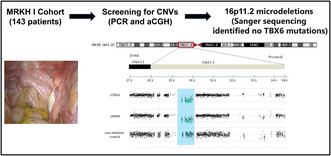

## 1 INTRODUCTION

Congenital uterine anomalies have been associated with infertility since different kinds of uterovaginal aplasia exists (Chan et al., 2011). The prevalence of uterine anomalies is around 3.5%–8.0% in women with infertility, with a trend toward higher incidence in the past decades (Chan et al., 2011; Nahum, 1998; Raga et al., 1997; Saravelos et al., 2008). Among all the uterine anomalies, Mayer–Rokitansky–Küster–Hauser (MRKH) syndrome (OMIM #277000) is a severe and rare disorder characterized by uterovaginal aplasia with a normal 46, XX karyotype (Chen et al., 2022). Despite aplasia of the uterus and upper vagina, the patients have normal tubes, ovaries, and secondary sexual characteristics. Nevertheless, infertility and inability of sexual intercourse largely affect patients' quality of life (Beisert et al., 2015). MRKH syndrome is generally diagnosed in female adolescents due to primary amenorrhea (Oppelt et al., 2012). The estimated incidence of MRKH syndrome is 1 in 5000 live female births (Herlin et al., 2016). Several studies suggested that the malformations associated with MRKH syndrome originate in abnormal formation or fusion of the Mullerian ducts during gestational weeks 4–12 (Oppelt et al., 2012). However, the pathogenesis of the syndrome is still unclear.

Clinically, classifications of MRKH syndrome resulted in two main subtypes. Type I MRKH syndrome is characterized with isolated Mullerian duct aplasia, including uterus, cervix, and 2/3 upper vagina (OMIM #277000), while Type II MRKH (OMIM #601076) occur as agenesis of Mullerian ducts with other extragenital malformations (Ledig & Wieacker, 2018). Among the additional abnormalities identified in type II MRKH patients, renal aplasia and skeletal malformation are frequently reported, whereas hearing impairment and heart defects are less observed (Fontana et al., 2017). Interestingly, the incidence of two subtypes of MRKH syndrome seem to be varied in different human populations. Five European studies of MRKH syndrome (1259 patients) have shown that type I MRKH accounted for 53.8% of the population diagnosed as MRKH syndrome (Creatsas et al., 2010; Deng et al., 2019; Herlin et al., 2016; Kapczuk et al., 2016). In Chinese patients, the rate of type I MRKH varies from 69.6% to 92.8% in epidemiological studies (Chen, Pan, et al., 2021; Deng et al., 2019; Pan & Luo, 2016). The higher rates of type I MRKH might be attributed to ethnic differences.

Though most of the cases are reported to be sporadic, familial clustering of MRKH syndrome has been observed in several families (Chen, Pan, et al., 2021). Through investigation of 10 families with several cases, Shokeir, 1978 proposed an autosome dominant inherited mode with sex‐limited inheritance and incomplete penetrance, which have been partly clarified in later researches (Fontana et al., 2017; Gervasini et al., 2010; Shokeir, 1978). Previous studies have also suggested copy number variations (CNVs), and candidate gene mutations are related to this syndrome (Bernardini et al., 2009; Cheroki et al., 2008; Ledig et al., 2011; McGowan et al., 2015; Nik‐Zainal et al., 2011; Sandbacka et al., 2013). Recurrent chromosome aberrations at 16p11.2, 17q12, and 22q11 regions have been noticed in MRKH syndrome despite low incidence rates. Additionally, a recent review summarized the reports of MRKH cases with CNVs and it revealed type II MRKH accounted for 78% of those cases (21 in 26 cases) (Chen et al., 2022). In 2011, Serena et al. first observed probands with 16p11.2 deletions in total of 112 patients, suggesting that CNVs at 16p11.2 are probably related to the etiology of MRKH syndrome (Nik‐Zainal et al., 2011). 16p11.2 deletions occur in 0.03‰ of all populations worldwide, it has been reported to be the risk factor of many diseases, such as congenital scoliosis, obesity, and autistic disorder, indicating its essential role in embryo development (Rosenfeld et al., 2010; Wu et al., 2015). In a Chinese discovery cohort involving 442 MRKH patients, four 16p11.2 deletions were identified in type II MRKH cases, whereas 16p11.2 CNVs was not observed in patients diagnosed with type I MRKH (Chen, Zhao, et al., 2021). It is interesting to note that few CNVs was identified in Chinese type I MRKH cases although it accounted for the majority. Therefore, CNVs in Chinese type I MRKH patients call for more research based on various methods to answer this question.

Through screening for the regions related to CNVs identified and key genes involved in the development of Müllerian duct, several candidate causative genes have been proposed to elucidate the pathogenesis of MRKH syndrome. Müllerian duct is formed under the stimulus of rapidly withdraw of anti‐Mullerian hormone (AMH) (Oppelt et al., 2005; Resendes et al., 2001; Zenteno et al., 2004). *HOX* gene family and *WNT* gene family are the receptors of AMH (Lalwani et al., 2008; Timmreck et al., 2003). Previous studies showed that *HOX* genes did not have any mutations in MRKH patients (Ekici et al., 2013; Lalwani et al., 2008). However, Pontecorvi, P et al. has found that the expression of protein kinase X (PRKX) is increased in vaginal keratinocytes from MRKH patients and PRKX overexpression significantly affect the expression of *HOX* genes (Pontecorvi, Bernardini, et al., 2021; Pontecorvi, Megiorni, et al., 2021). In addition, De novo or rare *WNT4* genes mutations were only identified in MRKH patients with hyperandrogenism. *LHX1* functions as a transcription factor regulating protein–protein interactions. Ledig et al. suggested that heterozygous mutations of *LHX1* might be a cause for a subgroup of MRKH patients (Ledig et al., 2012). Moreover, with lost function of *Lhx1* (KO), female mice models had the phenotype of renal malformation and the lack of reproductive duct (Kobayashi et al., 2004). Among the candidate causative genes, *TBX6*, which locates in 16p11.2 and encodes a conserved transcription factor functioning in early embryogenesis, seems to be an important candidate gene reported to be associated with MRKH syndrome (Nik‐Zainal et al., 2011). There have been three studies which identified one potential pathogenic missense and two splice site mutation in total among MRKH patients (Chen, Pan, et al., 2021; Sandbacka et al., 2013; Tewes et al., 2015). It is also reported carrying variants in both *TBX6* and *LHX1* or a CNV in combination with *TBX6* variants might play a role in the etiology of Mullerian aplasia (Sandbacka et al., 2013). Interestingly, since only some of the cases have been attributed to genetic anomalies, other presumption of pathologic mechanisms such as epigenetics and somatic mutations have been suggested to play a role in Mullerian aplasia (Buchert et al., 2022; Hentrich et al., 2020). And the application of techniques including whole genome sequencing (WGS) and RNA‐seq is expected to provide new clues hopefully (Buchert et al., 2022; Hentrich et al., 2020; Pan et al., 2019).

In this study, we recruited 143 type I MRKH Han patients in Shanghai of Eastern China, using array‐based comparative genomic hybridization (aCGH) and Quantitative PCR to identify CNVs and candidate causative gene.

## 2 MATERIALS AND METHODS

### 2.1 Participants and ethical approval

This study was approved by the institutional review boards of International Peace Maternity and Child Health Hospital of China welfare institute (No. GKLW 2017‐101) and Fudan University. All the participants were diagnosed as type I MRKH, without skeleton, urinary tract, and other malformation. This study was approved by the institutional review boards of International Peace Maternity and Child Health Hospital of China welfare institute and Fudan University. We obtained written informed consent from the participants (those who were ≥18 years of age at the time of enrollment) or their guardians (for participants who were <18 years of age). Peripheral blood was collected from each participants and stored at −80°C before analysis.

### 2.2 Quantitative PCR

The aim of producing quantity PCR is to detect the 16p11.2 deletion in 143 MRKH patients. We designed three pairs of primers, two pairs located in 16p11.2 region (Primer A‐F/R and Primer B‐F/R) and the other one located out of the region (Primer 1‐F/R). A sample with 16p11.2 deletion and another sample with non‐deletion previously confirmed by the human genome CGH microarrays were selected as the positive and negative controls respectively. Those samples derived from previous research in our university and have been described before (Wu et al., 2015; Yang et al., 2019). Once the ΔCT value of a sample gets close to the positive control, it indicates that the template amounts of primer 1‐FR and A/B‐FR are quite different and the possibility of 16p11.2 deletion is high. On the contrary, the possibility of 16p11.2 deletion is little. ΔCT value is calculated by [(A + B)/2−C]. In our study, ΔCT value of the positive control was 1.807. We got two samples with ΔCT value very close to 1.807 (ΔCT value: 1.905 and 1.647) and identified by aCGH. The experiment was performed following the protocol in the Data S1.

### 2.3 Array comparative genomic hybridization

The array comparative genomic hybridization experiment identifying 16p11.2 deletion was following Agilent oligonucleotide CGH protocol (version 6.0) principles. High‐quality gDNA samples were extracted from patients' blood samples with the concentration >90 μL/mL, A260/A280 ratio of 1.8 to 2.0 and A260/A230 > 1.0. DNAs were fragmented by Alu and Rsa enzyme. Then DNAs were labeled by Agilent SureTag DNA Labeling Kit. Normal control DNA from the kit was labeled by Cy3‐dUTP fluorescence dyes, while the patient DNA was labeled by Cy5‐dUTP fluorescence dyes. Each pair of control and patient DNA were mixed and hybridized onto Agilent SurePrint G3 human 1 × 1 M microarray at 65°C for 40 hours.

### 2.4 DNA sequencing

The entire *TBX6* gene and its∼1 kb upstream region were amplified using long‐range PCR for Sanger sequencing. We used the same experimental conditions as described previously to screen the 143 patients diagnosed with type I MRKH (Wu et al., 2015).

### 2.5 Statistical analysis

Fisher's exact test was used to investigate different prevalence of 16p11.2 microdeletions between 143 gathered MRKH patients and 500 control persons.

## 3 RESULTS

**FIGURE 1:**
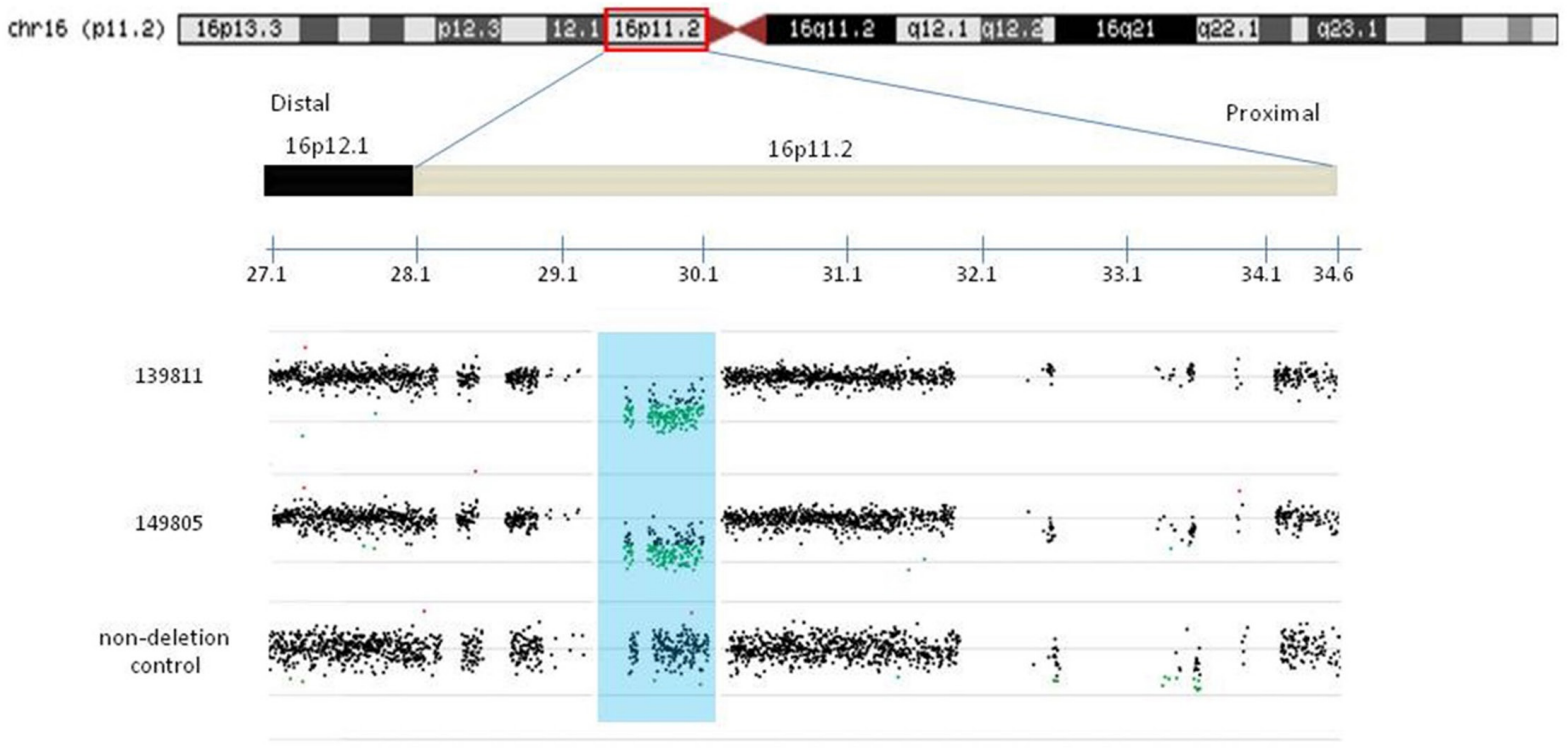
High‐density aCGH identified 16p11.2 microdeletions in two subjects (139,811 and 149,805). The oligos indicating copy number loss were shown in green.

This study reports the CNV results based on qPCR analysis and aCGH technique in Chinese Han population of 143 type I MRKH patients. We conducted qPCR to screen for the 16p11.2 deletion in 143 type I MRKH syndrome patients. Subsequently, we used CGH microarrays to perform a genome wide analysis of CNVs in 33 type I MRKH syndrome patients including two patients with deletions in 16p11.2 identified by qPCR previously. As shown in Figure 1, CNV analysis reveals deletions in 16p11.2 region in two patients, both with the deletion length of 1,001,182 bp. Additionally, we analyzed variants within *TBX6* across all 143 samples using Sanger sequencing. However, no damaging mutations were detected. Additional information on the ΔCT value of the two patients can be found in the Data S1. Both patients have typical secondary sex characteristics with a 46, XX karyotype (Table 1). The two patients exhibited normal levels of reproductive hormones, including estradiol, progesterone, total testosterone, prolactin, follicle‐stimulating hormone, and luteinizing hormone, as well as normal kidney function. X‐ray examinations revealed no skeletal and pulmonary deformities. Neither patient had a family history of MRKH. The specific clinical characteristics of the two patients in whom the deletions were identified are described below.

**TABLE 1:** Information of the two subjects identified with 16p11.2 microdeletion.

Sample number	Gender	Age	Karyotype	Associated anomalies	Population
139,811	Female	26	46, XX	Type I MRKH^a^	Chinese Han
149,805	Female	24	46, XX	Type I MRKH^a^	Chinese Han

^a^ Type I MRKH refers to absence of uterus and vagina with no anomalies in other systems.

### 3.1 Patient 1

**FIGURE 2:**
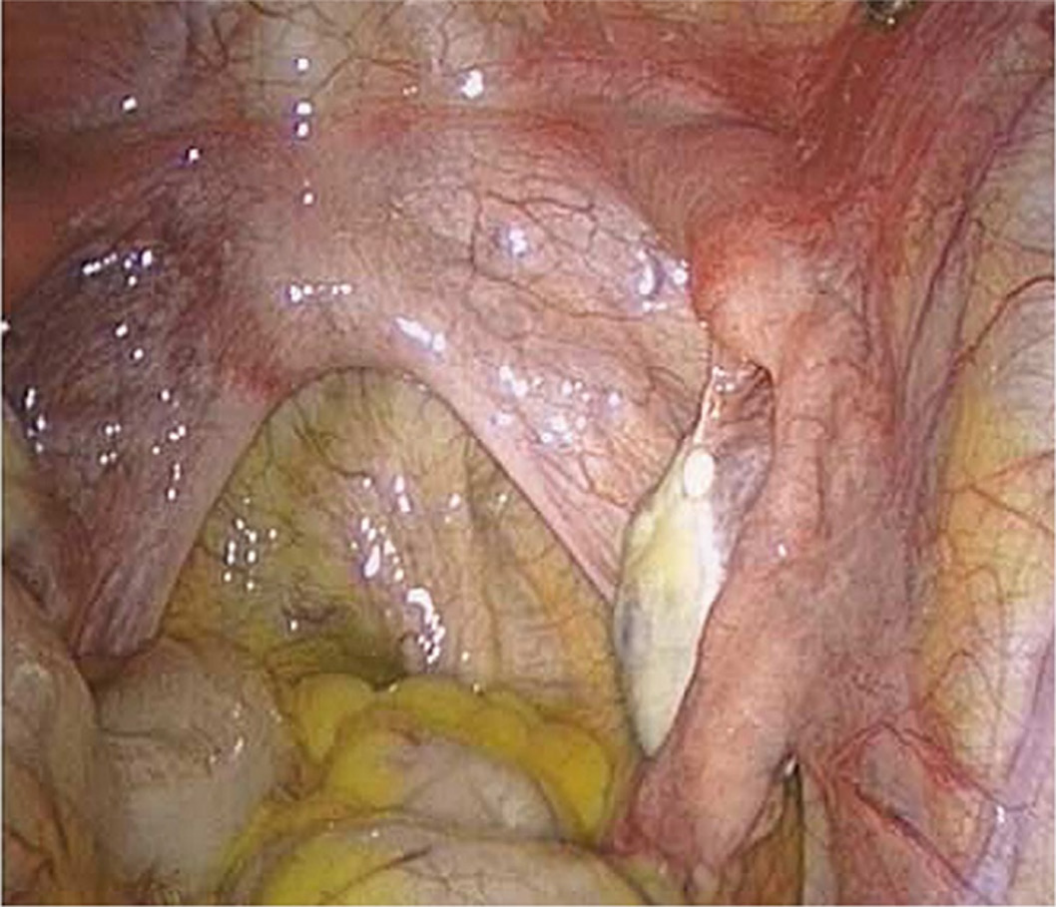
Pelvic condition of the patient by laparoscopy. The image presented absence of uterus, normal fallopian tubes and ovaries.

This patient was diagnosed with congenital absence of uterus and upper vagina due to primary amenorrhea and recurring lower quadrant abdominal pain. Normal uterine appendages were confirmed through laparoscopy (Figure 2) and reproductive hormone measurement. The depth of the patient's vagina was measured to be 5 cm, while the sonogram revealed the left ovary to be 37 × 19 mm and the right ovary to be 34 × 20 mm in size. Ultrasound examinations did not detect any abnormalities in the ureter, bladder, or kidneys. The patient came to our observation for fertility counseling at the age of 26 years. She was 150 cm tall and weighed 42.5 kg.

### 3.2 Patient 2

This 24‐year‐old women underwent artificial vaginoplasty in our hospital. She was diagnosed as MRKH (at age of 21 years) for congenital absence of uterus and vagina. Physical examinations revealed normal breasts, labia, and distribution of pubic hair. Intravenous pyelography did not detect any abnormalities. The patient's mother presented a later onset of menstruation.

## 4 DISCUSSION

The etiology and pathogenic mechanism of MRKH syndrome remains poorly understood. A number of studies aimed to investigate the genetic causes of MRKH syndrome, focusing on CNVs and candidate genes involving *TBX6, AMH*, *LHX1*, *WNT*, and *HOX*. In the past years, aCGH technique and next‐generation sequencing (NGS)‐based gene panel made it convenient to discover the cryptic chromosome aberrations responsible for congenital malformations (Pontecorvi, Bernardini, et al., 2021; Thomson et al., 2023; Triantafyllidi et al., 2022). Recurrent aberrations in 17q12, 22q11.2, 1q21.1, and 16p11.2 have been reported to be related with MRKH syndrome (Ledig et al., 2011; Nik‐Zainal et al., 2011; Sundaram et al., 2007). However, no monogenetic factor of MRKH has been identified yet in addition to *WNT4* mutations observed in patients with hyperandrogenism, which suggests the need of more researches in this field (Fontana et al., 2017). To our best knowledge, this is the first CNVs analysis performed in Chinese MRKH syndrome based on aCGH. Despite a lot of algorithms have been developed to detect CNVs from sequencing data, high resolution version of aCGH is still an appropriate methodology for the analysis of chromosome aberrations in a cohort (Corallo et al., 2021; Retterer et al., 2015). The application of whole‐exome sequencing (WES) surely reduced related costs of sequencing while there is also deficiency considering the restricted detection in exomic space and the data processing itself (Rapti et al., 2022). Chromosome aberrations in MRKH patients has been only analyzed using WES data in the Chinese population (Chen, Zhao, et al., 2021). Similarly, they also found that recurrent aberrations in 16p11.2 are relatively frequent in the Chinese population. However, these aberrations were only identified in four patients diagnosed with type II MRKH syndrome. As a disease with high heterogeneity, genetic causes might play an important role which interact with other factors leading to different types or phenotypes of MRKH syndrome (Kyei‐Barffour et al., 2021). Type I MRKH take overwhelming majority of Chinese MRKH patients (Chen, Pan, et al., 2021; Deng et al., 2019; Pan & Luo, 2016). However, several chromosome aberrations, such as aberrations in 16p11.2, have not been reported in Chinese Type I MRKH patients. Thus, we designed the study using aCGH and Quantitative PCR to identify CNVs in Type I MRKH patients in Chinese Han population.

For the first time in this study, we report chromosome microdeletions in 16p11.2 identified in Chinese Type I MRKH patients. Notably, we did not observe any 16p11.2 deletions in 500 control people (*p* < 5*10−2 by unpaired *t* test), which is consistent with the reported frequency of copy number variants in the 16p11.2 region (1/30,000 worldwide) (Rosenfeld et al., 2010). Microdeletions at 16p11.2 were reported to be associated with various phenotypic consequences including obesity (Walters et al., 2010), autistic spectrum disorder (Kumar et al., 2008), and mental retardation (Ghebranious et al., 2007). As a hot spot pathogenesis region for some congenital genetic diseases, rearrangement in 16p11.2 region has attracted a lot of scientific focuses (McCarthy et al., 2009). There are 28 genes locate in this region, chromatin‐contacted genes were enriched in specific pathways like NOTCH signal pathway and PENT pathway. The possible mechanism of multiple organs simultaneous anomalies is that during the embryonic development, these systems and organs are derived from the same intermediate mesoderm (Duncan et al., 1979; Fontana et al., 2017). Recently, disruption of chromatin in this region has been proven to cause obesity (Loviglio et al., 2017). In 2015, Wu et al. explored the genetic mechanism of congenital scoliosis, *TBX6* null variants and a common hypomorphic allele will lead to 10% scoliosis patients (Wu et al., 2015). From previous researches, heterogeneity between human and mouse was obviously seen because Lim (Lhx1) homo‐knockout female mice showed uterus anomalies, while hetero‐knockout female mice showed normal reproductive system. Those results illustrate the complexity in detecting candidate genes related to microdeletions at 16p11.2.

The pathogenic mechanisms underlying the contribution of 16p11.2 deletion to MRKH syndrome development remain unclear. 16p11.2 region contains genes closely related to embryo development (McCarthy et al., 2009). Among those causative genes, *TBX6* is the most frequently suggested one, despite the relatively low incidence rates of its mutations in MRKH patients. The genes located in 16p11.2 region have been reported previously to be implicated in the development of paraxial mesoderm (White et al., 2003). One potential pathogenic missense and two splice site mutation within *TBX6* has been discovered in previous studies (Chen, Zhao, et al., 2021; Sandbacka et al., 2013; Tewes et al., 2015). However, in our study, no mutations in the *TBX6* gene were identified in the sample set. Other genes within this region might be involved in the association between 16p11.2 deletion and MRKH syndrome development, but determining the exact causative gene(s) is difficult and the lack of whole‐genome sequencing data has limited our further genetic analysis. Additionally, we are curious about whether the deletion in the 16p11.2 region affects the expression levels of various genes within the region. We are looking forward to the large‐scale applications of whole genome sequencing (WGS) or long‐read NGS to give us more evidence in this field given the fact that deficiencies exist in current common‐used techniques.

The results of our study are consistent with the findings observed in European patients and hold implications for genetic counseling among women with type I MRKH syndrome (Nik‐Zainal et al., 2011; Sandbacka et al., 2013). As natural fertilization is impossible for females with MRKH, large pedigrees for genome linkage analysis are scarce (Fontana et al., 2017). In vitro fertilization and surrogacy are feasible ways to reproduce offspring using their own eggs as their ovaries are functioning normal. Nevertheless, concerns about transmitting the congenital abnormality to offspring of the patients must be addressed through accurate genetic counseling before pregnancy to explore the risks of uterine aplasia and other congenital abnormalities. Another limitation of this study is that we did not know whether the 16p11.2 deletion was inherited or de novo as the blood samples of the patients' family members were not available. Further investigation is still warranted.

In conclusion, enrichment of copy number variant at 16p11.2 (almost 1/70) in Chinese Han MRKH patients highlights the genetic pathogenic role of 16p11.2 deletion in MRKH syndrome. We did not find any mutations in *TBX6* gene in our samples, however, we cannot negate the impact of *TBX6* in Mullerian duct development. Our results identify 16p11.2 deletion in Chinese MRKH I patients for the first time and this is the first study to conduct CNV analysis based on aCGH in Chinese MRKH patients. Further investigations are necessary to identify causative genes and molecular mechanisms for MRKH syndrome.

## AUTHOR CONTRIBUTIONS

Kaizhen Su and Han Liu performed experiments and drafted the manuscript. Han Liu and Xiaoqun Ye were responsible for the processing of samples. Hangmei Jin, Zhenwei Xie, and Chunbo Yang contributed to the recruitment of patients. Daizhan Zhou helped conduct the data analysis. Hefeng Huang and Yanting Wu contributed to the design of the study, interpretation of the data, and final approval of the version to be published. All the authors approved the final version of the manuscript.

## FUNDING INFORMATION

This research is supported by National Key Research and Development Program of China (2021YFC2700701, 2022YFC2703505), National Natural Science Foundation of China (8211101588, 82088102, 82171686), CAMS Innovation Fund for Medical Sciences (2019‐I2M‐5‐064), the International Science and Technology Collaborative Fund of Shanghai (18410711800), Program of Shanghai Academic Research Leader (20XD1424100), Natural Science Foundation of Shanghai (20ZR1463100), Shanghai Clinical Research Center for Gynecological Diseases (22MC1940200), Shanghai Urogenital System Diseases Research Center (2022ZZ01012) and Shanghai Frontiers Science Research Base of Reproduction and Development.

## CONFLICT OF INTEREST STATEMENT

The authors declare that they have no competing interests.

## ETHICS STATEMENT

This study was approved by the institutional review boards of International Peace Maternity and Child Health Hospital of China welfare institute (No. GKLW 2017‐101) and Fudan University. All procedures performed in this study involving human participants were in accordance with the ethical standards of the institutional and/or national research committee and with the 1964 Helsinki declaration and its later amendments or comparable ethical standards.

## CONSENT FOR PUBLICATION

Informed consent to report individual patient data was obtained by the participants (those who were ≥18 years of age at the time of enrollment) or their guardians (for participants who were <18 years of age).

## Supporting information


Data S1.

## Data Availability

The data that support the findings of this study are available from the corresponding author upon reasonable request. Primers and protocol are shown in the Data S1.
