# Supplementary material for: Recurrent human 16p11.2 microdeletions in type I Mayer–Rokitansky–Küster–Hauser (MRKH) syndrome patients in Chinese Han population
Source: Mol Genet Genomic Med. 2023 Oct 3;12(1):e2280. doi: 10.1002/mgg3.2280 (PMC10767395; doi:10.1002/mgg3.2280)
Supplement: Supplementary file 1 — Data S1. [file MGG3-12-e2280-s001.docx]

**Additional file 1. Primers design and cycling conditions for quantitative PCR**

Primers for the quantitative PCR assay for 16p11.2 deletion in human subjects.

| Primer | Sequence |
| --- | --- |
| Primer 1-F | 5'-GGGGAAGGAACTTACATGAC-3' |
| Primer 1-R | 5'-TCGTGTTTCCCTGTTGTACC-3' |
| Primer A-F | 5'-GGTCTAAGCCACACACTAAC-3' |
| Primer A-R | 5'-TGAGTTTAGGGACCAATCTA-3' |
| Primer B-F | 5'-GCTGCCAGTATGTGACCGAGA-3' |
| Primer B-R | 5'-GGGTGGAGGAGAGGATAGGG-3' |

Cycling conditions of the quantitative PCR assay for 16p11.2 deletion in human subjects.

| Program | Cycles | Analysis  Mode | Target  (°C) | Hold  (hh:mm:ss) | Acquisitions  (per °C) | Acquisition  mode |
| --- | --- | --- | --- | --- | --- | --- |
| Pre-incubation | 1 | None | 95 | 00:02:00 | --- | None |
| Amplification | 40 | Quantification | 95 | 00:15:00 | --- | None |
|  |  |  | 60 | 00:01:00 | --- | None |
|  |  |  | 95 | 00:15:00 | --- | None |
| Melting curve | 1 | Melting curves | 60 | 00:15:00 | 10 | Continuous |
|  |  |  | 95 | 00:15:00 | --- | None |

After the reaction, SDS 2.4.1(Applied Biosystems) was used to analysis the data.

Cycle threshold (Ct) value of the quantitative PCR assay.

| **Primer** | **Positive control** | | **139811** | | **149805** | | **Negative control** | |
| --- | --- | --- | --- | --- | --- | --- | --- | --- |
|  | **Three technical repetition** | **Mean value** | **Three technical repetition** | **Mean value** | **Three technical repetition** | **Mean value** | **Three technical repetition** | **Mean value** |
|  |  |  |  |  |  |  |  |  |
| **A** | 26.39508 | 26.3968285 | 26.532896 | 26.42187433 | 26.191856 | 26.17977433 | 25.303305 | 25.34083233 |
|  | 26.839542 |  | 26.380135 |  | 26.168365 |  | 25.413502 |  |
|  | 26.398577 |  | 26.352592 |  | 26.179102 |  | 25.30569 |  |
|  |  |  |  |  |  |  |  |  |
| **B** | 26.612223 | 26.64424733 | 26.687977 | 26.594332 | 26.585085 | 26.52352133 | 25.51669 | 25.44640933 |
|  | 26.680506 |  | 26.511492 |  | 26.466501 |  | 25.329147 |  |
|  | 26.640013 |  | 26.583527 |  | 26.518978 |  | 25.493391 |  |
|  |  |  |  |  |  |  |  |  |
| **1** | 24.697775 | 24.71364667 | 24.597595 | 24.60286333 | 24.866402 | 24.704892 | 24.575764 | 24.61843267 |
|  | 24.728077 |  | 24.595188 |  | 24.678259 |  | 24.689646 |  |
|  | 24.715088 |  | 24.615807 |  | 24.570015 |  | 24.589888 |  |
| **ΔCT value** |  | 1.80689125 |  | 1.905239833 |  | 1.646755833 |  | 0.775188167 |

ΔCT value is calculated by [(A+B)/2-C].
